# COVID-19 Vaccination Coverage Among Insured Persons Aged ≥16 Years, by Race/Ethnicity and Other Selected Characteristics — Eight Integrated Health Care Organizations, United States, December 14, 2020–May 15, 2021

**DOI:** 10.15585/mmwr.mm7028a1

**Published:** 2021-07-16

**Authors:** Cassandra Pingali, Mehreen Meghani, Hilda Razzaghi, Mark J. Lamias, Eric Weintraub, Tat'Yana A. Kenigsberg, Nicola P. Klein, Ned Lewis, Bruce Fireman, Ousseny Zerbo, Joan Bartlett, Kristin Goddard, James Donahue, Kayla Hanson, Allison Naleway, Elyse O. Kharbanda, W. Katherine Yih, Jennifer Clark Nelson, Bruno J. Lewin, Joshua T.B. Williams, Jason M. Glanz, James A. Singleton, Suchita A. Patel

**Affiliations:** ^1^Immunization Services Division, National Center for Immunization and Respiratory Diseases, CDC; ^2^Leidos, Inc., Atlanta, Georgia; ^3^CDC COVID-19 Response Team; ^4^Division of Healthcare Quality Promotion, National Center for Emerging and Zoonotic Infectious Diseases, CDC; ^5^Kaiser Permanente Vaccine Study Center, Oakland, California; ^6^Marshfield Clinic Research Institute, Marshfield, Wisconsin; ^7^Center for Health Research, Kaiser Permanente Northwest, Portland, Oregon; ^8^HealthPartners Institute, Minneapolis, Minnesota; ^9^Harvard Pilgrim Health Care Institute, Boston, Massachusetts; ^10^Biostatistics Unit, Kaiser Permanente Washington Health Research Institute, Seattle, Washington; ^11^Research and Evaluation, Kaiser Permanente Southern California, Pasadena, California; ^12^Ambulatory Care Services, Denver Health, Denver, Colorado and University of Colorado School of Medicine, Aurora, Colorado; ^13^Institute for Health Research, Kaiser Permanente Colorado, Denver, Colorado; Department of Epidemiology, Colorado School of Public Health, Aurora, Colorado

COVID-19 vaccination is critical to ending the COVID-19 pandemic. Members of minority racial and ethnic groups have experienced disproportionate COVID-19–associated morbidity and mortality (*1*); however, COVID-19 vaccination coverage is lower in these groups (*2*). CDC used data from CDC’s Vaccine Safety Datalink (VSD)* to assess disparities in vaccination coverage among persons aged ≥16 years by race and ethnicity during December 14, 2020–May 15, 2021. Measures of coverage included receipt of ≥1 COVID-19 vaccine dose (i.e., receipt of the first dose of the Pfizer-BioNTech or Moderna COVID-19 vaccines or 1 dose of the Janssen COVID-19 vaccine [Johnson & Johnson]) and full vaccination (receipt of 2 doses of the Pfizer-BioNTech or Moderna COVID-19 vaccines or 1 dose of Janssen COVID-19 vaccine). Among 9.6 million persons aged ≥16 years enrolled in VSD during December 14, 2020–May 15, 2021, ≥1-dose coverage was 48.3%, and 38.3% were fully vaccinated. As of May 15, 2021, coverage with ≥1 dose was lower among non-Hispanic Black (Black) and Hispanic persons (40.7% and 41.1%, respectively) than it was among non-Hispanic White (White) persons (54.6%). Coverage was highest among non-Hispanic Asian (Asian) persons (57.4%). Coverage with ≥1 dose was higher among persons with certain medical conditions that place them at higher risk for severe COVID-19 (high-risk conditions) (63.8%) than it was among persons without such conditions (41.5%) and was higher among persons who had not had COVID-19 (48.8%) than it was among those who had (42.4%). Persons aged 18–24 years had the lowest ≥1-dose coverage (28.7%) among all age groups. Continued monitoring of vaccination coverage and efforts to improve equity in coverage are critical, especially among populations disproportionately affected by COVID-19.

VSD is a collaboration between CDC’s Immunization Safety Office and eight integrated health care organizations in six U.S. states.^†^ VSD captures information on COVID-19 vaccine doses administered, regardless of where they are received, based on an automated search within the organizations’ facilities (outpatient and inpatient records) and external systems (e.g., health insurance claims and state or local immunization information systems). VSD data on administered COVID-19 vaccine doses were captured from December 14, 2020, when the Pfizer-BioNTech vaccine received Emergency Use Authorization in the United States,^§^ through May 15, 2021. Data were reported to CDC weekly beginning January 9, 2021. The analysis excluded 561 persons aged 16–17 years who received Janssen or Moderna vaccine, because persons in this age group were eligible for the Pfizer-BioNTech vaccine only during the study period.

Coverage with ≥1 COVID-19 vaccine dose was defined as the proportion of persons who received a first dose of Pfizer-BioNTech or Moderna COVID-19 vaccine, or 1 dose of Janssen COVID-19 vaccine. Full vaccination was defined as receipt of 2 doses of Pfizer-BioNTech or Moderna COVID-19 vaccine or 1 dose of Janssen COVID-19 vaccine. Coverage was estimated for persons who received ≥1 dose and persons who were fully vaccinated by racial or ethnic minority groups, age, sex, presence of a high-risk condition,^¶^ and COVID-19 illness history. Data on race and ethnicity were available for 88.3% of the VSD population; data on age, sex, high-risk condition, and history of COVID-19 illness were 100% complete. COVID-19 illness history was defined using an internal *International Classification of Diseases, Tenth Revision, Clinical Modification* (ICD-10-CM) diagnosis code indicating that the person had COVID-19** or a positive laboratory test result before vaccination. All analyses were performed using SAS (version 9.4; SAS Institute), and data were a census of all persons in the population (i.e., VSD); therefore, statistical tests were not performed to assess differences between groups. Absolute differences in coverage were calculated using White persons as the reference group (coverage among racial/ethnic group of interest minus coverage among White persons). This activity was reviewed by CDC and VSD sites and was conducted consistent with applicable federal law and CDC policy.^††^

During December 14, 2020–May 15, 2021, 9.6 million persons aged ≥16 years were continuously enrolled in VSD. The VSD population was 41.2% White, 6.5% Black, 24.2% Hispanic, 12.1% Asian, 3.3% non-Hispanic multiple or other race, 0.6% non-Hispanic Native Hawaiian or Other Pacific Islander, 0.3% non-Hispanic American Indian or Alaskan Native (AI/AN); race or ethnicity were unknown for 11.7%. By May 15, 2021, 48.3% of persons aged ≥16 years had received ≥1 COVID-19 vaccine dose, and 38.3% were fully vaccinated. 

Coverage with ≥1 COVID-19 vaccine dose was highest among Asian (57.4%) and White persons (54.6%) and lowest among Hispanic (41.1%) and Black persons (40.7%) (Table). Similar racial/ethnic variations were observed among fully vaccinated persons. Coverage with ≥1 dose increased weekly for all racial/ethnic minority groups during December 14, 2020–May 15, 2021 (Figure 1). Coverage as of Feb 13, 2021 was 8.2% for Hispanic persons, 9.3% for Black persons and 15.5% for Asian persons, while coverage as of May 15, 2021 was 41.1%, 40.7%, and 57.4% respectively. The absolute difference in coverage between White persons and all minority racial/ethnic groups generally increased over time, except Asian persons (Figure 1). The absolute percentage point difference in coverage between Black and White persons and between Hispanic and White persons increased over the study period from 6% (Black persons) and 8% (Hispanic persons) as of February 13, 2021 to 14% for both Black and Hispanic persons as of May 15, 2021.

**TABLE:** COVID-19 vaccination coverage among persons aged ≥16 years, by selected characteristics — Vaccine Safety Datalink, December 14, 2020─May 15, 2021

Characteristic	No. (%)*		
	VSD population	≥1 dose coverage^†^	Full vaccination coverage^§^
**Total**	**9,568,149 (100)**	**4,624,351 (48.3)**	**3,660,284 (38.3)**
**Age group, yrs**			
16–17	280,213 (2.9)	82,976 (29.6)	37,358 (13.3)
18–24	1,010,815 (10.6)	289,898 (28.7)	172,108 (17.0)
25–34	1,677,206 (17.5)	604,382 (36.0)	425,934 (25.4)
35–49	2,413,431 (25.2)	1,056,291 (43.8)	783,432 (32.5)
50–64	2,325,211 (24.3)	1,264,761 (54.4)	1,021,656 (43.9)
65–74	1,135,965 (11.9)	771,078 (67.9)	702,066 (61.8)
≥75	725,308 (7.6)	554,965 (76.5)	517,730 (71.4)
**Race/Ethnicity**			
White, non-Hispanic	3,942,027 (41.2)	2,153,286 (54.6)	1,779,948 (45.2)
Black, non-Hispanic	624,406 (6.5)	253,995 (40.7)	199,948 (32.0)
Hispanic/Latino	2,318,498 (24.2)	952,925 (41.1)	714,304 (30.8)
Asian, non-Hispanic	1,162,154 (12.1)	667,064 (57.4)	526,417 (45.3)
AI/AN, non-Hispanic	29,888 (0.3)	14,071 (47.1)	11,041 (36.9)
NH/PI, non-Hispanic	57,925 (0.6)	27,941 (48.2)	21,728 (37.5)
Multiple or other, non-Hispanic	312,524 (3.3)	153,614 (49.2)	119,954 (38.4)
Unknown	1,120,727 (11.7)	401,455 (35.8)	286,944 (25.6)
**Sex**			
Female	5,036,074 (52.6)	2,560,407 (50.8)	2,063,085 (41.0)
Male	4,532,075 (47.4)	2,063,944 (45.5)	1,597,199 (35.2)
**High risk for COVID-19 disease^¶^**			
No	6,624,221 (69.2)	2,746,712 (41.5)	2,031,841 (30.7)
Yes	2,943,928 (30.8)	1,877,639 (63.8)	1,628,443 (55.3)
History of COVID-19 disease**			
No	8,802,463 (92.0)	4,299,610 (48.8)	3,421,532 (38.9)
Yes	765,686 (8.0)	324,741 (42.4)	238,752 (31.2)

**Abbreviations:** AI/AN = American Indian or Alaska Native; ICD-10-CM = *International Classification of Diseases, Tenth Revision, Clinical Modification*; NH/PI = Native Hawaiian or Pacific Islander; VSD = Vaccine Safety Datalink.

* Percentages might not sum to expected values because of rounding.

^†^ At least 1 dose of COVID-19 vaccine is defined either as the first of 2 doses of Pfizer-BioNTech or Moderna vaccines, or 1 dose of the Janssen (Johnson & Johnson) vaccine from December 14, 2020–May 15, 2021.

^§^ Fully vaccinated is defined as receipt of both first and second dose of Pfizer-BioNTech or Moderna vaccines or receipt of 1 dose of Janssen (Johnson & Johnson) vaccine during December 14, 2020–May 15, 2021.

^¶^ All patients’ records from the outpatient and inpatient settings are screened (automated records review and chart review) for underlying medical conditions that increase the risk of severe COVID-19 illness using ICD-10-CM codes. https://www.cdc.gov/coronavirus/2019-ncov/need-extra-precautions/people-with-medical-conditions.html

** A combination of ICD-10 COVID-19 codes and internal medical diagnostics codes was used to identify persons with a history of COVID-19 illness. Patient records were also screened for positive laboratory tests for COVID-19 illness before receipt of vaccination. The new ICD-10-CM code came into effect on April 1, 2020. https://www.cdc.gov/nchs/data/icd/Announcement-New-ICD-code-for-coronavirus-3-18-2020.pdf

**FIGURE 1:**
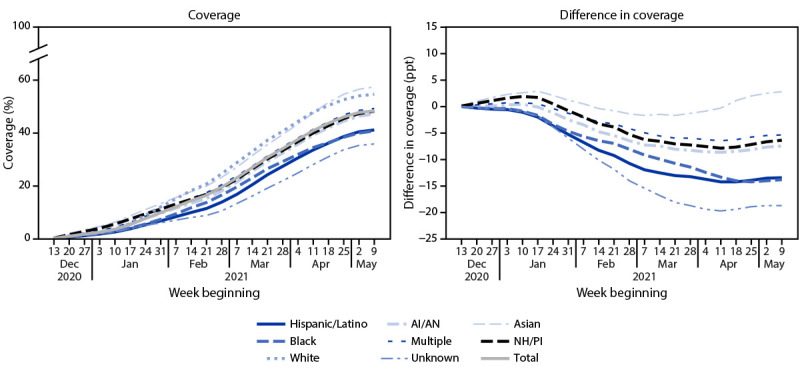
COVID-19 vaccination coverage* and difference in vaccination coverage^†^ among persons aged ≥16 years, by race/ethnicity^§^ and week — Vaccine Safety Datalink, United States, December 14, 2020–May 15, 2021 **Abbreviations:** AI/AN = American Indian or Alaska Native; NH = non-Hispanic; NH/PI = Native Hawaiian or Pacific Islander; ppt = percentage point. * At least 1 dose of COVID-19 vaccine is defined either as the first of 2 doses of Pfizer-BioNTech or Moderna vaccines, or a single dose of Janssen (Johnson & Johnson) vaccine during December 14, 2020–May 15, 2021. ^†^ Ppt difference in coverage from that in the White population (race/ethnicity coverage – NH, White coverage). ^§ ^Hispanic persons could be of any race. Black, White, Asian, AI/AN, NH/PI, and multiracial persons were non-Hispanic.

Coverage with ≥1 dose was highest among persons aged ≥75 years (76.5%) and decreased with age; coverage was 28.7% among persons aged 18–24 years and 29.6% among those aged 16–17 years. Coverage with ≥1 dose was higher among females (50.8%) than among males (45.5%) (Table). Coverage among women was higher than that among men stratified by age, except among those aged ≥75 years (Supplementary Table 1, https://stacks.cdc.gov/view/cdc/107670). Coverage with ≥1 dose was higher among persons with high-risk conditions (63.8%) than among persons without these conditions (41.5%), and among persons with no history of COVID-19 (48.8%) than among those with a history of COVID-19 (42.4%) (Table). Full vaccination coverage patterns were similar to ≥1-dose coverage by age, sex, high-risk conditions status, and history of COVID-19.

Asian persons had the highest coverage overall and among those with a high-risk condition (68.6%) or a history of COVID-19 illness (56.4%) (Figure 2). Among persons with and without high-risk conditions, Black persons had the lowest ≥1-dose coverage (55.6% and 30.4%, respectively).

Among persons aged ≥75 years, Black and Hispanic persons had similar ≥1-dose coverage to that of White and Asian persons; among younger age groups, ≥1-dose coverage among Black and Hispanic persons was lower than that among White and Asian persons (Supplementary Table 2, https://stacks.cdc.gov/view/cdc/107671). Variations in coverage were also observed by age (Supplementary Table 1, https://stacks.cdc.gov/view/cdc/107670) and vaccine type (Supplementary Table 3, https://stacks.cdc.gov/view/cdc/107672).

## Discussion

Among 9.6 million persons in the eight integrated health care organizations from six states included in the VSD, 48.3% of those aged ≥16 years had received ≥1 COVID-19 vaccine dose as of May 15, 2021, and 38.3% were fully vaccinated; cumulative ≥1-dose coverage increased steadily during the study period for all racial and ethnic minority groups during December 14, 2020–May 15, 2021. In the VSD population, ≥1-dose COVID-19 vaccination coverage among Asian and White persons was highest, and coverage among Hispanic and Black persons was lowest. This disparity has persisted and, generally, increased since December 2020, despite increasing vaccine availability and improved outreach efforts (*2*). Continued monitoring of vaccination coverage and efforts to improve equity in coverage are critical, especially among populations disproportionately affected by COVID-19. These efforts could include identifying and addressing potential barriers to access and improving vaccine acceptance, particularly among Hispanic and Black persons (*3*).

Coverage with ≥1 COVID-19 vaccine dose was higher among persons with a high-risk condition. This might be because such persons were prioritized for earlier allocation of COVID-19 vaccine. Persons with a history of COVID-19 had lower coverage than did persons who had not had COVID-19. This might indicate that these persons waited for some time after illness before seeking vaccination, or that they believed they might not need vaccination after recovering from COVID-19 (*4*).

Vaccination coverage increased with increasing age. Coverage was highest among older adults, likely because of earlier vaccine prioritization among this population compared with younger adults (*5*). Coverage with ≥1 COVID-19 vaccine dose was lowest among adults aged 18–24 years and among adolescents aged 16–17 years, for whom the only authorized COVID-19 vaccine is Pfizer-BioNTech. Persons aged 16–17 years are still generally under parental supervision, including with regard to medical and health decisions, which might have contributed to the higher coverage in this age group compared with that in those aged 18–24 years. Ensuring that vaccination is accessible and available in places where persons live and work could improve coverage, particularly among younger adults (*6*).

The findings in this report are subject to at least three limitations. First, because VSD collects data in six states within eight integrated health care organizations, these findings might not be generalizable to the U.S. population. However, previous research indicates that VSD estimates align closely with those from the U.S. population in many important demographic characteristics, including sex and racial/ethnic distributions (*7*). Second, vaccination coverage in the VSD population could be underestimated if vaccination status was not captured or identified for some persons who received vaccinations outside participating systems or state registry catchment areas. Finally, VSD data on race and ethnicity are 88.3% complete, which could limit interpretation of results.

COVID-19 vaccination coverage in the integrated health systems that comprise VSD is continuing to increase for all racial/ethnic groups, and coverage is higher among persons with medical conditions that place them at higher risk for severe COVID-19. However, racial and ethnic minority groups, including Black and Hispanic persons, continue to have lower coverage, and these gaps appear to have widened over time. Although vaccines are provided at no cost, persons who have economic challenges or language barriers, lack government-issued identification, or who face challenges because of distance or transportation to a vaccination site might experience barriers to vaccination (*8*). Efforts to address vaccine misinformation, barriers to access, and insufficient vaccine confidence, coupled with strategies to prioritize equity, could help increase coverage and reduce COVID-19 incidence, especially among populations disproportionately affected by the pandemic.

**Summary**

**What is already known about this topic?**

Non-Hispanic Black and Hispanic persons experience higher COVID-19–associated morbidity and mortality, yet COVID-19 vaccination coverage is lower in these groups.

**What is added by this report?**

As of May 15, 2021, 48.3% of persons identified in CDC’s Vaccine Safety Datalink aged ≥16 years had received ≥1 COVID-19 vaccine dose and 38.3% were fully vaccinated. Coverage with ≥1 dose was lower among non-Hispanic Black (40.7%) and Hispanic persons (41.1%) than among non-Hispanic White persons (54.6%); coverage was highest (57.4%) among non-Hispanic Asian persons.

**What are the implications for public health practice?**

Continued monitoring of vaccination coverage and efforts to improve equity in vaccination coverage are critical, especially among populations disproportionately affected by COVID-19.

**FIGURE 2:**
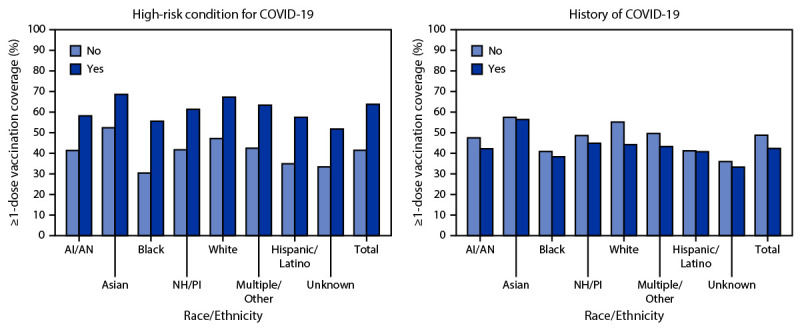
Coverage with ≥1 dose of COVID-19 vaccine* among persons aged ≥16 years, by race/ethnicity^†^ and having a high-risk condition for severe COVID-19 or history of COVID-19^§,¶^ — Vaccine Safety Datalink, United States, December 14, 2020–May 15, 2021 **Abbreviations:** AI/AN = American Indian or Alaska Native; ICD-10 = *International Classification of Diseases, Tenth Revision*; NH/PI = Native Hawaiian or Pacific Islander. * At least 1 dose of COVID-19 vaccine is defined either as the first of 2 doses of Pfizer-BioNTech or Moderna vaccines, or 1 dose of Janssen (Johnson & Johnson) vaccine during December 14, 2020–May 15, 2021. ^† ^Hispanic persons could be of any race. Black, White, Asian, AI/AN, NH/PI, and multiracial persons were non-Hispanic. ^§^ All patients’ records from the outpatient and inpatient settings are screened (automated records review and chart review) for underlying medical conditions that increase the risk of severe COVID-19 using ICD-10 codes. ^¶^ Medical diagnostic codes were used to identify persons with a history of COVID-19 illness. Patient records were also screened for positive lab tests for COVID-19 illness before vaccination. The new ICD-10-Clinical Modification code went into effect on April 1, 2020. https://www.cdc.gov/nchs/data/icd/Announcement-New-ICDcode-for-coronavirus-3-18-2020.pdf

## Footnotes

* https://www.cdc.gov/vaccines/covid-19/info-by-product/clinical-considerations.html, https://www.cdc.gov/vaccinesafety/ensuringsafety/monitoring/vsd/

† The eight participating VSD health care organizations are located in California (two sites), Colorado (two sites), Minnesota, Oregon, Wisconsin, and Washington, and monitor vaccine safety among 11.6 million insured persons. One additional site (in Massachusetts) provides subject matter expertise on vaccine safety research and surveillance.

§ As of May 2021, all adults aged ≥18 years can receive any of the three COVID-19 vaccines available in the United States via Emergency Use Authorization from the Food and Drug Administration, including Pfizer-BioNTech, Moderna, and Janssen (Johnson & Johnson); persons aged 16–17 years are eligible to receive the Pfizer-BioNTech COVID-19 vaccine.

¶ All patients’ records from outpatient and inpatient settings are screened by automated records review and chart review for underlying medical conditions that increase the risk for severe COVID-19, using ICD-10 codes. https://www.cdc.gov/coronavirus/2019-ncov/need-extra-precautions/people-with-medical-conditions.html

** A combination of ICD-10 COVID-19 codes and internal medical diagnostics codes were used to identify persons with a history of COVID-19. Patient records were also screened for positive laboratory test results indicating COVID-19 before receipt of vaccination. The new ICD-10-CM code came into effect April 1, 2020. https://www.cdc.gov/nchs/data/icd/Announcement-New-ICD-code-for-coronavirus-3-18-2020.pdf

†† 45 C.F.R. part 46.102(l)(2), 21 C.F.R. part 56; 42 U.S.C. Sect. 241(d); 5 U.S.C. Sect. 552a; 44 U.S.C. Sect. 3501 et seq.
